# From neurodevelopment to neurodegeneration: utilizing human stem cell models to gain insight into Down syndrome

**DOI:** 10.3389/fgene.2023.1198129

**Published:** 2023-05-30

**Authors:** L. Ashley Watson, Hiruy S. Meharena

**Affiliations:** Developmental and Cognitive Genomics Research Laboratory, Division of Biological Sciences, Section of Neurobiology, University of California, San Diego, La Jolla, CA, United States

**Keywords:** down syndrome, neurodevelomental disorders, neurodegenenerative diseases, human tissue, cell culture models, human stem cells

## Abstract

Down syndrome (DS), caused by triplication of chromosome 21, is the most frequent aneuploidy observed in the human population and represents the most common genetic form of intellectual disability and early-onset Alzheimer’s disease (AD). Individuals with DS exhibit a wide spectrum of clinical presentation, with a number of organs implicated including the neurological, immune, musculoskeletal, cardiac, and gastrointestinal systems. Decades of DS research have illuminated our understanding of the disorder, however many of the features that limit quality of life and independence of individuals with DS, including intellectual disability and early-onset dementia, remain poorly understood. This lack of knowledge of the cellular and molecular mechanisms leading to neurological features of DS has caused significant roadblocks in developing effective therapeutic strategies to improve quality of life for individuals with DS. Recent technological advances in human stem cell culture methods, genome editing approaches, and single-cell transcriptomics have provided paradigm-shifting insights into complex neurological diseases such as DS. Here, we review novel neurological disease modeling approaches, how they have been used to study DS, and what questions might be addressed in the future using these innovative tools.

## Introduction

We sit at an intersection of several complementary technologies including human pluripotent stem cells, genome editing tools, 2D and 3D culture of human stem cell-derived brain cell types, human-mouse chimeras, and single-cell transcriptomics. The combination of these technologies has permitted robust and rapid modeling of human neurological disorders and greater sensitivity to detect phenotypic differences, collectively enabling the field to uncover mechanistic details of human neurological diseases.

Down syndrome (DS), caused by triplication of human chromosome 21 (Lejeune et al., 1959), is a complex and multisystemic disorder (Antonarakis et al., 2020). Individuals with DS exhibit a range of clinical presentation on a spectrum of severity, including neurological, craniofacial, immunological, cardiac, gastrointestinal, cardiovascular, musculoskeletal, sleep, and behavioral abnormalities (Antonarakis et al., 2020). The most prominent consequence of trisomy 21 is intellectual disability (ID), and nearly all individuals with DS present mild to moderate ID (Gueant et al., 2005; Määttä et al., 2006). Moreover, DS is the greatest genetic risk factor for early-onset Alzheimer’s disease (AD) (Mann, 1988; McCarron et al., 2017; Snyder et al., 2020). For the purposes of this review, we will focus on the neurological aspects of DS, including neurodevelopmental and neurodegenerative pathologies.

Studies utilizing human postmortem tissue samples have provided a wealth of information related to the anatomical and cellular underpinnings of DS but these samples represent a clinical end-point that rarely allows for cause-and-effect analysis. A number of DS mouse models have been developed that enable mechanistic studies related to the consequence of trisomy 21 on neurodevelopment and brain function. However, the genes found on human chromosome 21 are distributed across a number of mouse chromosomes, which has challenged the generation of a mouse strain that effectively models the genetics of DS. While we have made great strides in this area, biochemical differences in protein sequence and physiology between humans and mice result in a failure of mouse models to accurately mimic human diseases with neurodevelopmental and neurodegenerative components like DS. In light of this, we still lack a detailed understanding of the molecular mechanisms that contribute to disease pathogenesis, which is underscored by the lack of effective therapeutics to alleviate the neurological features of DS.

Human stem cell technology has revolutionized disease modeling studies and methods continue to emerge that enhance our ability to generate the exquisite diversity of cell types observed in the human brain *in vitro*. Advances in 2D- and 3D-culture systems have paved the way for novel discoveries related to human brain development and function (Fernandes et al., 2021) and the mechanistic basis of neurological disorders (Vadodaria et al., 2020), providing exceptional platforms for scientific and therapeutic discovery (Bonaventura et al., 2021).

The neurological features of DS implicate all of the major brain cell types including neural progenitor cells (Stagni et al., 2018), neurons (Guidi et al., 2008; Bartesaghi, 2022), astrocytes (Zdaniuk et al., 2011; Ponroy Bally and Murai, 2021), oligodendrocytes (Olmos-Serrano et al., 2016), and microglia (Flores-Aguilar et al., 2020), and a subset of brain regions such as the cerebral cortex (Yun et al., 2021), hippocampus (Guidi et al., 2008; Koenig et al., 2021), cerebellum (Guidi et al., 2011), and retina (Haseeb et al., 2022). In this review, we will describe currently available methods for 2D- and 3D-differentiation of human stem cells into these cell types and brain regions, the work that has been done related to DS using stem cell modeling approaches, future questions that may be addressed with emerging technologies, and discuss the limitations of these models.

## Neurological features of Down syndrome

DS is the most common genetic cause of intellectual disability (ID) and impacts approximately 1 in 700 live births (Mai et al., 2019). ID in DS manifests as impairments in language acquisition, executive functioning (i.e., attention, self-control, future planning), and hippocampal-dependent declarative memory (Carlesimo et al., 1997; Vicari et al., 2000; Nadel, 2003; Cornish et al., 2007; Trezise et al., 2008; Lanfranchi et al., 2010; Costanzo et al., 2013; Godfrey and Lee, 2018; Dimachkie Nunnally et al., 2023), ranging from moderate to severe with intelligent quotient (IQ) scores between 30 and 70 (Gueant et al., 2005; Määttä et al., 2006). Individuals with DS are at a greater risk of neurological comorbidities such as epilepsy (Goldberg-Stern et al., 2001; Altuna et al., 2021), Alzheimer’s disease (Mann, 1988; McCarron et al., 2017; Snyder et al., 2020), and attention deficit hyperactivity disorder (ADHD) (Capone et al., 2006). In fact, nearly every individual with DS presents pathological features of Alzheimer’s disease by the age of 40, including amyloid plaques and neurofibrillary tangles of hyperphosphorylated Tau (pTau) (Wisniewski et al., 1985; Head et al., 2016; Snyder et al., 2020). Despite pathological presentation, some individuals with DS do not experience dementia, suggesting protective environmental or genetic resilience factors which still remain unknown.

Clinical features of DS present with varying severity and penetration in the population, despite the disorder consistently stemming from triplication of chromosome 21 (Letourneau et al., 2012). While some individuals with DS (2%–4%) are mosaic for trisomy 21 (T21), i.e., two or more genetically distinct cells develop from a single zygote resulting in a proportion of cells harboring T21 and a subset that are euploid (Papavassiliou et al., 2009), the vast majority of individuals with DS exhibit T21 in all cells. The variable penetrance of DS-related cognitive phenotypes has likely contributed to some of the discrepancies observed in human postmortem studies and concordance with model systems, i.e., mice and human cell culture (Manley and Anderson, 2019; Klein and Haydar, 2022).

**FIGURE 1:**
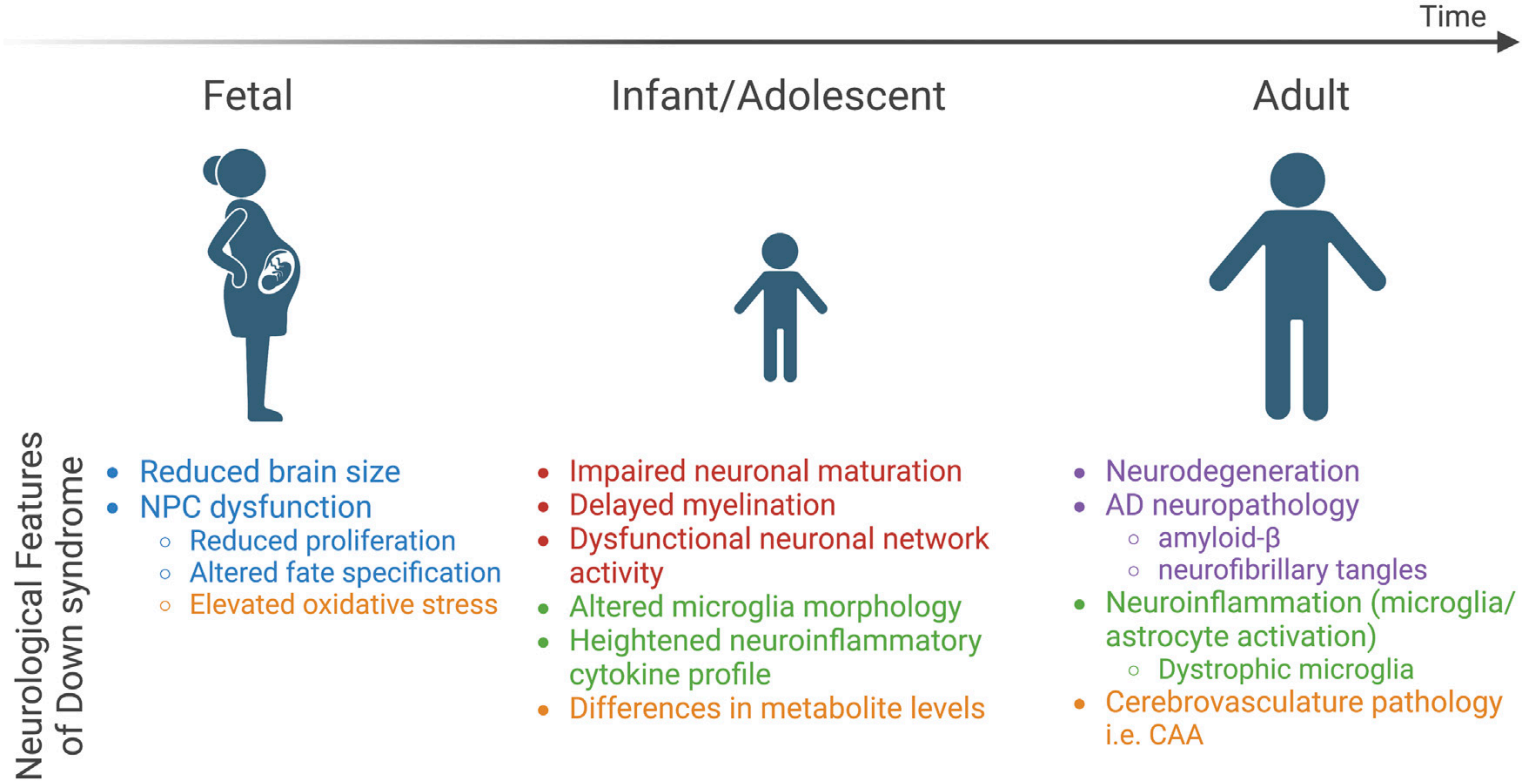
Neurological features of DS across the lifespan. Three developmental time points (fetal, infant/adolescent, and adult) are depicted and key neurological features identified in studies of postmortem DS brain tissue are highlighted below each time point. Text color indicates relation to five major categories: brain size and cell type composition changes (blue), neuronal network activity (red), neuroinflammatory phenotypes (green), neurodegenerative pathology (purple), and metabolic dysfunction (orange). NPCs, neural progenitor cells; AD, Alzheimer’s disease.

In spite of this, some features are consistent, including the fact that individuals with DS exhibit atypical neurodevelopment with effects that persist into adulthood (Klein and Haydar, 2022). The most prominent neurological features of DS include reduced brain size, altered cell type composition, abnormal neuronal communication and network activity, pathological hallmarks of Alzheimer’s disease, and neuroinflammation, which are all believed to contribute to altered brain function that results in intellectual disability and an increased risk of early-onset dementia (Figure 1).

### Altered brain size and cell type composition in Down syndrome

Postmortem analysis of DS brains indicates brain alterations as early as 15 gestational weeks (gw) (Guihard-Costa et al., 2006) and reduced volume by 21–23 gw (Golden and Hyman, 1994; Patkee et al., 2020) (Figure 1). By adulthood, the brains of individuals with DS are approximately 20% smaller than neurotypical individuals when corrected for body size (Hamner et al., 2018). The cerebral cortex, hippocampus, and cerebellum are regions particularly impacted by trisomy 21, and reduced volume is correlated with neuronal hypocellularity in these structures (Pinter et al., 2001; Guidi et al., 2008; Guidi et al., 2011). The mechanism of reduced neuronal number remains unresolved; however, studies implicate neural progenitor cells (NPC) dysfunction (Figure 1). NPCs give rise to the majority of brain cell types including neurons and macroglia (astrocytes and oligodendrocytes) at specific time windows during development. Reduced NPC proliferation in the mid-to late-gestational period (Contestabile et al., 2007; Guidi et al., 2008; Stagni et al., 2018; Baburamani et al., 2020) and altered differentiation dynamics have been observed in the DS brain (Guidi et al., 2008; Guidi et al., 2018). While overall neuronal number is reduced, excitatory neurons appear to be more sensitive to trisomy 21 (Guidi et al., 2018; Stagni et al., 2020), although specific subpopulations of inhibitory interneurons (i.e., calretinin-positive cells) are also reduced in DS (Guidi et al., 2018; Giffin-Rao et al., 2022). Overall, the altered frequency of subtype-specific neuronal populations is believed to result in an excitation-inhibition imbalance, a candidate mechanism for intellectual disability in DS and other disorders with underlying intellectual disability (Fernandez and Garner, 2007). It is important to note that this hypothesis primarily stems from observations made in DS mouse models (Fernandez et al., 2007; Kleschevnikov et al., 2012; Valbuena et al., 2019; Zorrilla de San Martin et al., 2020), and human data in this regard is lacking. Recent single-cell RNA-sequencing (scRNA-seq) from postmortem DS brain samples corroborated these findings and, based on the transcriptional signature of the inhibitory interneurons that occurred at higher frequency in the DS brain relative to neurotypical individuals, suggested that interneurons arising from the caudal ganglionic eminence (CGE) rather than those arising from the medial ganglionic eminence (MGE) are elevated in DS (Palmer et al., 2021). Further, some studies indicate skewed ratios of glial lineage cells such as astrocytes relative to neuronal number (Becker et al., 1991; Mito and Becker, 1993; Griffin et al., 1998; Colombo et al., 2005; Guidi et al., 2008; Lu et al., 2011; Guidi et al., 2018) and others suggest impaired oligodendrocyte differentiation (Olmos-Serrano et al., 2016). Collectively, these findings point to NPCs as a key cell type impacted in DS, and the cerebral cortex, hippocampus, and cerebellum as brain structures that are particularly vulnerable to trisomy 21.

### Abnormal neuronal network activity in Down syndrome

Precise regulation of neuronal ensembles is critical to support fundamental cognitive processes such as learning and memory and relies on the appropriate generation of neuronal subtypes, neuronal maturation and synaptogenesis, as well as the modulation and sculpting of neuronal circuits by other brain cell types (oligodendrocytes, astrocytes, and microglia). At the cellular level, the process of neuronal maturation is altered in infants and adolescents with DS (Suetsugu and Mehraein, 1980; Takashima et al., 1981; Becker et al., 1986; Ferrer and Gullotta, 1990; Becker et al., 1991; Takashima et al., 1994) and cortical cultures derived from fetal tissue indicates impaired neuronal differentiation and reduced neurite length (Bahn et al., 2002; Bhattacharyya et al., 2009) (Figure 1). Both synaptic density and dendritic complexity are reduced in children with DS, and this phenotype is exacerbated in an age-dependent manner with the appearance of neurodegenerative pathologies (Petit et al., 1984; Becker et al., 1986; Takashima et al., 1989; Becker et al., 1991). Alongside these changes in neuronal maturation, the frontal and temporal lobes of the brains of individuals with DS exhibit delayed myelination and reduced white matter content, leading to reduced axonal insulation (myelin), which alters neurotransmission (Wisniewski and Schmidt-Sidor, 1989; Becker et al., 1991; Koo et al., 1992; Ábrahám et al., 2012). Indeed, synaptic plasticity, in particular, long term potentiation (LTP), has been shown to be impaired in individuals with DS (Battaglia et al., 2008). At the network level, altered spontaneous brain activity has been reported in DS (Bartesaghi, 2022; Cañete-Massé et al., 2022) alongside increased functional connectivity (Csumitta et al., 2022) and network synchrony (Anderson et al., 2013), which correlates with lower IQ in individuals with DS (Anderson et al., 2013). In terms of resting state networks, the default mode network (DMN), composed of the medial prefrontal cortex, medial temporal lobe, and the posterior cingulate cortex, is engaged in internally focused tasks including autobiographical memory retrieval and future planning, which is believed to provide a means for adaptive behavior (Buckner et al., 2008). In individuals with DS, the DMN exhibits a more complex pattern of connectivity that is inversely correlated with cognitive performance (Figueroa-Jiménez et al., 2021) and quality-of-life values (Carbó-Carreté et al., 2020), indicating that it may represent an indicator of overall wellbeing in individuals with DS. Event-related potentials (ERPs) are voltage changes in the brain that represent the summed postsynaptic potentials generated in synchrony in large neuron populations that are processing a specific input and can be measured in humans to examine the effects of specific inputs on brain activity (Woodman, 2010). Prolonged latency in auditory, visual, and somatosensory ERPs have been reported in children and young adults with DS (Ferri et al., 1994; Ferri et al., 1995; Ferri et al., 1996; Karrer et al., 1998; Chen and Fang, 2005), suggesting impaired central processing of these input signals. Together, human studies indicate deficits in neuronal maturation that is associated with impaired myelination, altered neuronal circuit activity, and aberrant brain function in DS.

### Neuroinflammatory phenotypes in individuals with Down syndrome

Individuals with DS are a high-risk group in terms of susceptibility to and severity of infection. Both innate and adaptive immune systems are implicated in the immune dysregulation observed in DS (Huggard et al., 2020). Chronic neuroinflammation, which refers to activation of immune cells within the central nervous system (CNS), including microglia and astrocytes, and release of pro-inflammatory cytokines, is associated with cognitive impairment in neurodevelopmental disorders and plays an important role in the development and progression of AD (Leng and Edison, 2021; Liu et al., 2022). In both plasma and brain tissue samples, proteomic analyses indicate elevated levels of both pro- and anti-inflammatory cytokines in DS (Sullivan et al., 2016; Zhang et al., 2017; Flores-Aguilar et al., 2020; Huggard et al., 2020) (Figure 1). Morphological changes consistent with activation have been reported to occur in DS microglia and astrocytes (Griffin et al., 1989; Stoltzner et al., 2000; Colombo et al., 2005; Streit et al., 2009; Xue and Streit, 2011; Kanaumi et al., 2013; Flores-Aguilar et al., 2020; Martini et al., 2020; Hendrix et al., 2021; Palmer et al., 2021). While astrocytes haven’t been well studied in the human DS brain, microglial activation has been reported in infants and adolescents with DS (Flores-Aguilar et al., 2020). Recent scRNA-seq of DS brain indicated an elevated number of microglia with transcriptional profiles that resemble an activated microglial state in both young (<36 years old, without AD pathology) and old (>36 years old) individuals (Palmer et al., 2021). Aged individuals with DS exhibit a unique microglial morphology characteristic of a dystrophic state, which is associated with process swelling and beading, cell rupture, and ferritin expression, and suggestive of microglial senescence (Streit et al., 2009; Xue and Streit, 2011). Taken together, individuals with DS experience general immune system dysfunction and neuroinflammatory phenotypes, yet how these features are caused by T21 and relate to clinical presentation of DS such as intellectual disability, neurodevelopmental delay, and early-onset dementia remains unclear.

### Metabolic dysfunction in Down syndrome

At the cellular and systemic level, energy metabolism is altered in individuals with DS (Dierssen et al., 2020). This includes mitochondrial alterations, increased oxidative stress, as well as impaired glucose and lipid metabolism that culminates in reduced energy production and cellular dysfunction. Mitochondrial dysfunction associated with oxidative stress is considered to be an inherent feature of DS as a number of different cell types and tissues exhibit mitochondrial abnormalities in individuals with DS (Valenti et al., 2018). In particular, DS NPCs show elevated reactive oxygen species (ROS) levels (Esposito et al., 2008) and DS neurons display elevated oxidative stress-induced apoptosis *in vitro* (Busciglio and Yankner, 1995) (Figure 1)*.* Mitochondrial fragmentation and altered function is observed in DS fibroblasts, neurons, and astrocytes (Helguera et al., 2013). Metabolomics analysis of urine and plasma samples from individuals with DS show altered levels of metabolites involved in the Krebs cycle, glycolysis, and oxidative phosphorylation (Caracausi et al., 2018) (Figure 1), which are central metabolic processes related to mitochondria metabolism. To date, the best candidate for mitochondrial dysfunction in DS is *SOD1* Collectively, studies to date support the hypothesis that trisomic cells have impaired mitochondrial oxidative phosphorylation resulting in elevated oxidative stress and a shift towards glycolysis in trisomic cells to meet energy demands.

Emerging evidence suggests that metabolic defects are a risk factor for cognitive impairment in DS (Caracausi et al., 2018; Head et al., 2018; Vacca et al., 2019). The brain has an incredibly high metabolic demand compared to other tissues, and neurons in particular consume approximately 75%–80% of energy produced in the brain (Howarth et al., 2012). To meet these high-energy demands, the brain requires a continuous supply of oxygen and nutrients from the blood stream, which is accomplished through adequate perfusion of the brain with vasculature. Perfusion changes in specific brain regions including the temporal, parietal, and occipital lobes has been observed in individuals with DS (Kao et al., 1993). Cerebrovascular pathology such as cerebral amyloid angiopathy (CAA) is a hallmark of AD (Viswanathan and Greenberg, 2011) and DS-AD (Ikeda et al., 1994; Head et al., 2017) (Figure 1), is linked to cognitive decline in dementia. Further, studies suggest a direct connection between metabolic defects, amyloid deposition, and dementia, as insulin levels can impact the production and deposition of amyloid-ß in the brain (Wei et al., 2021) and brain insulin resistance can predict development of AD in DS (Tramutola et al., 2020). The available evidence indicates that DS cells show an intrinsic metabolic defect linked to mitochondrial dysfunction and systemically individuals with DS exhibit altered levels of key metabolites. These metabolic issues may be exacerbated by cerebrovascular changes in the brains of individuals with DS and could be related to intellectual disability and cognitive impairment in DS.

Metabolic dysfunction is a hallmark of DS and current evidence suggests that T21 causes impaired mitochondrial function. However, our understanding of how this manifests in the brain, an organ with incredibly high metabolic demand, is not well understood. Further, different brain cell types rely on mitochondrial energy metabolism to different extents, and the consequence of glycolysis usage in these cell types is not well understood.

## Rodent models of Down syndrome

Given that human postmortem tissue typically represents a pathological endpoint, it lacks the temporal resolution and the ability for experimental manipulations that model systems provide. Over the past several decades a number of mice strains have been generated to model DS (Herault et al., 2017). Human chromosome 21 (HSA21) has three orthologous regions on mouse chromosomes 10, 16, and 17. The largest syntenic region of HSA21 lies on mouse chromosome 16 (102/158 homologous protein coding genes), and the first DS mouse model was trisomic for Mmu16 (Ts16) (Reeves et al., 1986). Ts16 mice were lethal prior to birth, limiting analyses to embryonic period and calling into question how well these mice truly model DS. Improvements in transgenic rodent modeling resulted in the generation of over a dozen strains with varying degrees of similarity to the human disorder. The most widely utilized model is Ts65Dn (Davisson et al., 1993), resulting from translocation of the distal region of Mmu16 onto the centromeric region of Mmu17. Ts65Dn mice survive into adulthood and recapitulate several key features of DS including neurodevelopmental phenotypes as well as learning and memory deficits (Reeves et al., 1995). However, the Ts65Dn model has exhibited phenotypic drift over time (Shaw et al., 2020), causing the field to question the utility of this model moving forward. In the 25 years that Ts65Dn was regarded as the most effective rodent model of DS, studies demonstrated altered NPC proliferation and differentiation resulting in neuronal hypocellularity, altered cell type composition, microglia activation, and decreased synaptic density in the cortex, hippocampus, and cerebellum (Insausti et al., 1998; Baxter et al., 2000; Belichenko et al., 2004; Olson et al., 2004; Roper et al., 2006; Chakrabarti et al., 2007; Aziz et al., 2018; Illouz et al., 2019). More recently, cloning of the q arm of HSA21 into a species-specific artificial chromosome containing the native centromeric region has enabled generation of mouse (TcMAC21) (Kazuki et al., 2020) and rat (TsHSA21rat) (Kazuki et al., 2022) DS models. These models have the most comparable gene dosage (93% of protein-coding HSA21 genes) to that observed in humans with trisomy 21 compared to other rodent DS models. Initial characterization of the TcMAC21 mouse indicates reduced cerebellar volume and behavioral changes consistent with those observed in previous mouse models of DS (Kazuki et al., 2020). However, while the triplicated chromosome harbors human chromosome 21 genes, the other two copies of chromosome 21 are mouse, and therefore the phenotypes observed in this model may not fully recapitulate human clinical features.

Notably, overexpression of human transgenes harboring disease-causing variants is required to model neurodegenerative pathologies (i.e., AD-associated amyloid pathology and tauopathy) in rodents due to species-specific genetic differences. This has made rodent modeling of DS-AD a challenge, as complex breeding strategies are required to obtain rodents harboring a genetic background that is capable of presenting a pathology similar to what is observed in humans.

Rodent models of human neurological disorders are only as useful as their ability to recapitulate disease phenotypes. While several DS mouse strains model some aspects of the disorder and have provided significant insight into the cellular and molecular mechanisms underlying those specific phenotypes, there does not exist a single model that exhibits all of the clinical features observed in humans with DS [reviewed in (Klein and Haydar, 2022)]. There are also key differences between rodent and human brain cell types, such as astrocytes (Oberheim et al., 2009) and microglia (Gosselin et al., 2017), in terms of transcriptional profiles and functional characteristics, suggesting that the study of human brain cell types is more appropriate to gain insights into human neurological disease.

## Human induced pluripotent stem cell models of Down syndrome

Human iPSCs represent an exceptional model system to study the molecular and cellular underpinnings of neurological disorders. Any cell type can theoretically be differentiated from iPSCs, which can be derived from any individual using non-invasive procedures. More advanced 3-dimensional (3D) culture systems enable co-culture of different brain cell types and patterning factors can be used to generate brain region-specific organoids. While these systems do not allow for behavioral read-outs like rodent models provide, progress in the area of human-mouse chimeras may enable these types of studies. Below, we review current iPSC-derived brain culture systems and highlight application of these systems in the context of DS. Finally, we propose how emerging iPSC-derived culture technologies can help expand our knowledge of the cellular and molecular mechanisms underlying neurological deficits in DS.

It has been almost 3 decades since the first reports demonstrating successful conversion of differentiated somatic human cells into stem cells using the reprogramming factors OCT4, SOX2, KLF4, and cMYC (OSKM), referred to as induced pluripotent stem cells (iPSCs) (Takahashi et al., 2007; Yu et al., 2007; Park et al., 2008a). Human iPSCs completely revolutionized stem cell research as they evaded ethical implications of human embryonic stem cell models. The first DS iPSC lines were reported in 2008 (Park et al., 2008b) and were compared to age- and sex-matched iPSCs from neurotypical individuals. However, challenges in iPSC variability and reproducibility plagued early disease modeling studies (Guenther et al., 2010; Hu et al., 2010; Bock et al., 2011; Anderson et al., 2021). High levels of phenotypic variability were observed in cell lines generated from “healthy controls,” presumably due to the large degree of naturally occurring genetic variation that exists in the general population.

The barrier of genetic variability has largely been overcome with the generation of isogenic lines through improved single-cell cloning methods and novel genome editing techniques such as CRISPR-Cas systems. Genome editing enables the precise introduction or correction of pathogenic variants into endogenous genetic loci, and subcloning of parental and modified lines results in the generation of isogenic lines that are identical except for the genetic change of interest. In the context of DS, the first isogenic lines were described by several groups in 2012 and 2013 (Li et al., 2012; Maclean et al., 2012; Weick et al., 2013) wherein spontaneous loss of the third copy of chromosome 21 enabled subcloning of disomic and trisomic iPSCs from the same parental iPSC line. Alternatively, cells isolated from individuals with mosaic DS were subcloned to generate isogenic pairs (Weick et al., 2013; Murray et al., 2015), and lines have been established from twins discordant for T21 (Hibaoui et al., 2014). Alternatively, Li et al. (2012) utilized a genetic approach to select for disomic cells by integrating a thymidine kinase transgene into the third copy of chromosome 21 and treating cells with gancyclovir to select against T21.

Isogenic lines provide an ideal platform with which to conduct iPSC studies since they mitigate effects of genetic diversity and serve as critical controls for *in vitro* differentiation experiments. Several studies have utilized isogenic DS iPSC lines to study various aspects of the disorder *in vitro.* In the majority of cases, fibroblasts or blood cells were isolated from individuals with DS for reprogramming. More recently, a group demonstrated the utility of reprogramming urine-derived epithelial cells into iPSCs (Teles e Silva et al., 2023), representing a less invasive technique to obtain somatic cells from affected individuals. Regardless of how the iPSCs are derived, studies indicate that trisomic iPSCs have lengthened cell cycle kinetics compared to their isogenic disomic counterparts, with an elevated proportion of interphase (G1) cells and a concomitant reduction in S- and G2/M-phase cells (Li et al., 2012). Despite this, we and others have reported minimal differences between control (euploid) and trisomic iPSCs in terms of self-renewal, pluripotency, and transcriptional profiles (Park et al., 2008b; Li et al., 2012; Maclean et al., 2012; Meharena et al., 2022) (Table 1). Beyond a subset of upregulated genes located on chromosome 21, T21 iPSCs exhibited minimal transcriptional changes of genes located on other chromosomes (Li et al., 2012; Weick et al., 2013; Meharena et al., 2022). This is in stark contrast to the transcriptional and epigenomic changes observed in iPSC-derived neural cell types, reviewed below. The mechanism underlying cell type-specific epigenomic and transcriptional differences induced by T21 remains unknown.

**TABLE 1:** DS-related phenotypes observed in iPSC-derived cell culture systems and remaining questions that can be addressed with the emerging technology.

Culture system	Phenotype(s) observed in DS iPSC-derived system(s)	Select remaining question(s)
Induced pluripotent stem cells (iPSCs)	• Lengthened cell cycle Li et al. (2012) • Minimal impact on pluripotency or transcriptional signature Park et al. (2008b); Li et al. (2012); Maclean et al. (2012); Meharena et al. (2022)	What is the consequence of altered cell cycle kinetics on pluripotency and/or fate specification?
Neural progenitor cells (NPCs)	• Reduced proliferation Weick et al. (2013); Hibaoui et al. (2014); Meharena et al. (2022) • Altered differentiation Shi et al. (2012a); Briggs et al. (2013); Jiang et al. (2013); Lu et al. (2013); Weick et al. (2013); Chen et al. (2014); Hibaoui et al. (2014); Hirata et al. (2020); Klein et al. (2021) • Oxidative stress and mitochondrial abnormalities Sobol et al. (2019); Mollo et al. (2021); Prutton et al. (2022); Prutton et al. (2023) • Senescence-like chromatin architecture and transcriptional signature Meharena et al. (2022)	What is the mechanism underlying altered fate specification and senescence-like signature? • *NPC cultures*
Neurons	• Synaptic deficits Weick et al. (2013); Hibaoui et al. (2014) • Oxidative stress-induced apoptosis Weick et al. (2013); Sobol et al. (2019) • Dysregulation protein homeostasis and endoplasmic reticulum (ER) stress Hirata et al. (2020) • AD pathology: A*β* and pTau Shi et al. (2012a); Dashinimaev et al. (2017); Toshikawa et al. (2021)	Why are some neuronal subtypes more vulnerable to T21? • *Neuronal subtype-specific cultures*
Oligodendrocytes	n/a	Does T21 impact oligodendrocyte differentiation and/or myelination? • *Oligodendrocyte-neuron-astrocyte-microglia co-cultures and/or oligodendrocyte-enriched organoids*
Astrocytes	• Altered calcium signaling dynamics Mizuno et al. (2018) • Elevated oxidative stress Chen et al. (2014); Mizuno et al. (2018) • Induce neuronal toxicity Chen et al. (2014); Araujo et al. (2018); Mizuno et al. (2018) • Perturbed migration Ponroy Bally et al. (2020)	What is the consequence of T21 on astrocyte function? • *Improved astrocyte differentiation methods; co-culture systems*
Cerebral organoids	• AD pathology: A *β* and pTau Gonzalez et al. (2018); Zhao and Haddad (2022); Campbell et al. (2023); Czerminski et al. (2023) • Altered proliferation and delayed neurogenesis Tang et al. (2021); Li et al. (2022); Campbell et al. (2023) • Altered neuronal network activity and impaired excitatory-to-inhibitory balance Foliaki et al. (2021)	What is the mechanism underlying altered NPC fate specification? • *Unguided cerebral organoids*
Brain region-specific organoids • Cortical • Hippocampal • Ventral forebrain • Cerebellar • Retinal	• Altered NPC proliferation and delayed neurogenesis in cortical organoids Gonzalez et al. (2018); Tang et al. (2021); Li et al. (2022); Zhao and Haddad (2022); Campbell et al. (2023); Czerminski et al. (2023) • Ventralized medial ganglionic eminence (MGE)-like organoids showed elevated interneuron differentiation Xu et al. (2019)	Examination of brain region-specific vulnerability in DS. • *Cerebellar, hippocampal, retinal, and cortical organoids* Analysis of neuronal circuit activity. • *Cortical-hippocampal assembloids*
Blood-brain barrier and vascularized organoids	n/a	How does T21 impact the BBB and what is the mechanism of cerebrovasculature pathology? • *Vascularized organoids*
Microglia (iMGLs)	n/a	How does T21 impact myeloid lineage differentiation and microglia function? • *iMGL-engrafted organoids*
Human-mouse chimeras	• Altered cortical progenitor differentiation and neuronal functional deficits Real et al. (2018) • Impaired interneuron migration Huo et al. (2018) • Microglia activation and aberrant synaptic pruning Jin et al. (2022) • Dystrophic microglia in response to pathological Tau Jin et al. (2022)	Examination of the neuro-immune interplay in DS. • *Xenotransplanted microglia* How do T21 neurons integrated into specific circuits impact behavior? • *Xenotransplanted NPCs*

### Human stem cell differentiation approaches

**FIGURE 2:**
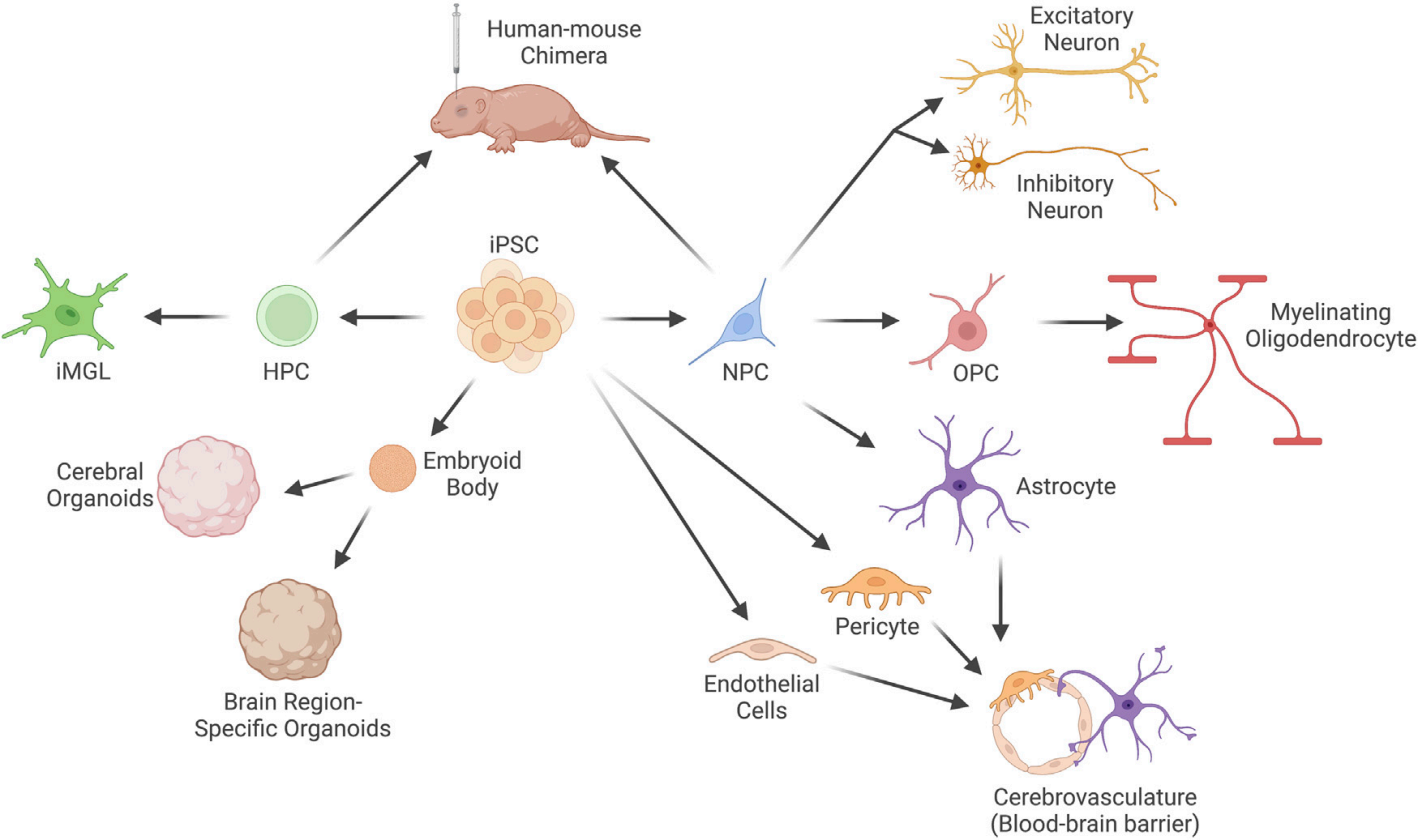
Current techniques to differentiate human induced Pluripotent Stem Cells (iPSCs) into brain cell types. Human iPSCs can be differentiated into neural progenitor cells (NPCs), which are the progenitor cell for neurons (both excitatory and inhibitory), myelinating oligodendrocytes and their progenitors (oligodendrocyte progenitor cells, OPCs), and astrocytes. iPSCs can also be differentiated into the other two primary cell types of the blood-brain barrier (BBB): endothelial cells and pericytes, which can be co-cultured with astrocytes to recapitulate the BBB *in vitro*. Embryoid bodies can be formed from aggregates of iPSCs and cultured in unguided or guided conditions to generate cerebral organoids or brain region-specific organoids. Microglia-like cells (iMGLs) of the myeloid lineage can be differentiated from hematopoietic progenitor cells (HPCs) *in vitro*. Alternatively, HPCs or NPCs can be engrafted into the brain of immunodeficient mice to generate human-mouse chimeras.

Numerous approaches to differentiate human iPSCs into the different brain cell types and structures exist, and additional methods continue to be developed that better model brain development and function *in vitro* (Figure 2)*.* The majority of differentiation protocols currently rely on removal of factors required for maintenance of the stem cell state and introduction of patterning molecules into the cell culture medium (we will refer to these methods as factor-mediated differentiation). These protocols attempt to mimic *in vivo* differentiation programs and, in general, are quite successful in guiding cell fate choices. This is especially true for progenitor populations, such as neural progenitor cells (NPCs), which can be generated by dual suppression of mothers against decapentaplegic (SMAD) inhibition in approximately 3 weeks (Chambers et al., 2009). However, for more terminally differentiated cell types, these approaches suffer from low purity, inefficiency, and prolonged culture periods. For instance, neuronal differentiation typically requires 8–12 weeks and generates a mixed population of excitatory and inhibitory neurons at variable ratios, as well as some remaining neural progenitor cells and off-target differentiation towards glial lineages (Burke et al., 2020; Lin et al., 2021).

More rapid approaches have since been developed to differentiate cells using a similar approach to the initial groups that described iPSCs (Deng et al., 2021). Overexpression of cell type-specific transcription factors that act to define cell state is sufficient to produce certain cell types, such as excitatory neurons (Zhang et al., 2013), oligodendrocytes (Ehrlich et al., 2017), and microglia (Dräger et al., 2022) (we will refer to these methods as overexpression-mediated differentiation). In general, these techniques are capable of producing differentiated cell types from iPSCs (or, in some cases, somatic cell types such as fibroblasts (Vierbuchen et al., 2010) in a much shorter timeframe. In some cases, such as with *Neurogenin 2* (*NGN2*) overexpression (Zhang et al., 2013), mature neurons can be generated in a couple of weeks compared to months with traditional growth factor-mediated approaches. However, overexpression is more technically challenging than media supplementation, and the most ideal transcription factor “cocktails” have not yet been identified for the majority of brain cell types. As our knowledge of the molecular players that define subtype-specific cell states grows, along with advances in our ability to generate stable transgenic cell lines, we will undoubtedly begin to improve these methods. Both methods of differentiation have been utilized to generate brain cell types from individuals with DS (Table 1), and in the following text we will highlight the approach used and the implication of their findings to our understanding of DS.

## iPSC-derived neural progenitor cells (NPCs)

Neural progenitor cells (NPCs) are a heterogeneous population of cells that give rise to the exquisitely diverse set of neurons and glia of the central nervous system. One of the most efficient and common methods to differentiate human stem cells into NPCs is through dual SMAD inhibition (Chambers et al., 2009). SMAD signaling plays a critical role in neural induction by destabilizing the TGF/activin- and Nanog-dependent pluripotency network, suppressing mesoendodermal fates through activin and nodal inhibition, and promoting neuralization of primitive ectoderm through bone morphogenic protein (BMP) inhibition. NPCs generated by dual SMAD inhibition generally resemble dorsal forebrain NPCs, and can be further patterned through addition of ventralization factors such as sonic hedgehog (SHH) or neuronal or glial subtype-specific factors such as retinoic acid (RA), BMPs, and Wingless/Integrated molecules (WNTs).

NPCs differentiated from DS iPSCs exhibit reduced proliferation (Hibaoui et al., 2014; Murray et al., 2015; Meharena et al., 2022), altered neuronal differentiation (Shi et al., 2012a; Briggs et al., 2013; Jiang et al., 2013; Lu et al., 2013; Weick et al., 2013) and a bias towards glial differentiation (Briggs et al., 2013; Chen et al., 2014; Hibaoui et al., 2014) (Table 1). Recent studies in isogenic DS NPCs indicated perturbed lineage specification due to altered patterning factor responses alongside aberrant WNT (Giffin-Rao et al., 2022) and SHH (Klein et al., 2021) signaling. NPCs derived from DS iPSCs also display dysfunctional mitochondrial metabolism (Mollo et al., 2021; Prutton et al., 2023), elevated ROS (Mollo et al., 2021; Prutton et al., 2022), as well as dysregulation of oxidative phosphorylation and glycolysis genes (Sobol et al., 2019) (Table 1). Whole-genome analysis of genome folding using chromosome conformation capture (Hi-C) demonstrated that DS NPCs exhibit global changes in chromatin structure that resemble cellular senescence (Meharena et al., 2022) (Table 1). DS NPCs also exhibit a senescence-like transcriptional signature and several cellular hallmarks of senescent cells such as reduced lamin B1 expression, abnormal heterochromatin distribution, and upregulation of senescence-associated secretory phenotype (SASP) molecules, in addition to reduced proliferation and migration. Treatment of DS NPCs with the senolytic drug cocktail of dasatinib and quercetin (DQ) restored proliferation and migration of DS NPCs and alleviated the transcriptional changes associated with T21 (Meharena et al., 2022). This study underscores the utility of taking an unbiased approach to investigate the cellular and molecular impacts of T21.

Taken together, given that NPCs give rise to the major cell types of the brain besides microglia, results from DS iPSC-derived NPCs suggest progenitor cell dysfunction that could explain the clinical finding of reduced brain size and altered frequency of specific brain cell types such as neuronal (excitatory versus inhibitory) and glial (astrocyte and oligodendroglial) populations. It remains unclear why NPCs are more vulnerable to T21 than other cell types (i.e., iPSCs) and how exactly T21 alters fate specification of NPCs. Future studies utilizing both 2D monocultures of NPCs as well as organoids (see 3D models section below) will be useful in addressing these remaining questions.

## iPSC-derived neurons

Neurons represent the primary functional unit of the brain; they use electrical and chemical signals to relay information between different brain regions and between the brain and the rest of the body. Neuronal circuits in the central nervous system are composed of two major categories of neurons: excitatory and inhibitory neurons. In general, excitatory neurons propagate network activity while inhibitory neurons regulate network activity through negative feedback. There exists an enormous variety of different excitatory and inhibitory neuronal subtypes, which are classified based on characteristics such as morphology, transcriptional profile, receptor expression, and neurotransmitter utilization. Heterogeneous or homogeneous neuronal populations can be differentiated from NPCs using passive, factor-mediated, or overexpression-mediated approaches. Passive differentiation relies on the propensity of NPCs to spontaneously differentiate to neurons and, to a lesser extent, astrocytes, upon removal of factors that promote NPC proliferation. Overexpression-mediated differentiation typically relies on overexpression of the neuronal transcription factor *NGN2*, which results in neuronal cultures in under 2 weeks (Zhang et al., 2013). Factor-mediated approaches rely on removal of fibroblast growth factor 2 (FGF2) (Shi et al., 2012b) and can utilize RA, ascorbic acid, adenoside 3′,5′-cyclic monophosphate (cAMP), brain-derived neurotrophic factor (BDNF), and glial cell line-derived neurotrophic factor (GDNF) (Bardy et al., 2015). This combination generates a mixed population of excitatory and inhibitory neurons, with an overall greater proportion of excitatory neurons, in a couple of months. It is also possible to enrich for inhibitory neurons by starting with ventrally patterned NPCs and culturing those cells in the presence of SHH and the SHH agonist purmorphamine (Liu et al., 2013; Huo et al., 2018). These conditions support differentiation to an inhibitory neuronal fate; however, the diversity of interneuron subtypes is challenging to recapitulate in *in vitro* monolayer conditions. Methods have been developed to promote specific subtypes using different growth factors. For instance, basal forebrain cholinergic neurons can be differentiated from ventrally patterned NPCs using nerve growth factor (NGF) (Nilbratt et al., 2010), and somatostatin- and parvalbumin-expressing interneurons can be generated with overexpression of LIM homeobox 6 (LHX6) (Yuan et al., 2018). In all neuronal differentiation cultures, there exists variable levels of residual NPCs and/or differentiation into off-target cell types such as different neuronal subtypes or astrocytes. Proliferative cell types such as NPCs and astrocyte precursors can be selected against using anti-mitotic agents such cytosine arabinoside (AraC).

With DS iPSCs, passive differentiation approaches have been shown to result in a differentiation shift towards astrocytes at the expense of neurons (Chen et al., 2014; Hibaoui et al., 2014; Hirata et al., 2020). Directed differentiation of DS NPCs to mixed cortical neurons is associated with impaired maturation and synaptic deficits (Weick et al., 2013; Hibaoui et al., 2014) (Table 1). Further, elevated oxidative stress (Weick et al., 2013; Dashinimaev et al., 2017; Sobol et al., 2019; Toshikawa et al., 2021), increased cell death, and pathological hallmarks of AD such as elevated amyloid-ß and hyperphosphorylated tau (Shi et al., 2012a; Dashinimaev et al., 2017; Toshikawa et al., 2021) have also been observed in DS iPSC-derived neurons (Table 1). Transcription factor-driven (*NGN2*) neuronal differentiation resulted in increased apoptosis of T21 neurons that was associated with dysregulated protein homeostasis and upregulation of endoplasmic reticulum stress pathway (Hirata et al., 2020). Directed differentiation of DS NPCs towards GABAergic interneuron fate revealed a less complex morphology of DS interneurons, altered subtype-specific differentiation, and impaired migratory ability (Huo et al., 2018).

Collectively, studies in iPSC-derived neurons differentiated in 2D have recapitulated some key features observed in the DS brain including altered differentiation, synaptic deficits, and AD-associated pathologies. Impaired differentiation and maturation of DS neurons could explain some clinical features of DS such as neuronal hypocellularity, altered excitation/inhibition balance, and reduced synaptic plasticity. In the future, it will be of interest to differentiate DS iPSCs into specific neuronal subtypes that exhibit selective vulnerability in the disorder, such as basal forebrain cholinergic neurons. Nevertheless, monolayer neuronal cultures are limited in their ability to faithfully recapitulate brain development and suffer from a lack of additional cell types that provide trophic, metabolic, and physical support, including astrocytes, oligodendrocytes, and microglia. Below, we review literature related to differentiation approaches for these other brain cell types as well as more complex 3D culture models of neurodevelopment and brain function such as organoids.

## iPSC-derived glia

After decades of focus on neuron-centric mechanisms of neurological diseases including DS and AD, recent studies have shifted to incorporate a more comprehensive understanding of the multiple different brain cell types including macro- and micro-glia.

## iPSC-derived astrocytes

Astrocytes are the most abundant cell type in the human brain. During development, they provide support for neuronal survival, axon and dendrite outgrowth, and synaptogenesis. In the adult brain, astrocytes provide physical, energetic, metabolic, and trophic support to neurons and other brain cell types (sidoryk-wegrzynowicz et al., 2011; Weber and Barros, 2015; Bélanger et al., 2011). While early studies of neurological disorders primarily focused on neuronal cells, a growing appreciation of the other cell types of the brain, such as astrocytes, has led to an increase in reports of glial function in brain development and disease (Khakh and Sofroniew, 2015). Our understanding of the consequence of T21 on astrocytes remains limited. Analysis of postmortem brain tissue suggests increased astrocyte frequency (Guidi et al., 2008) and altered morphology (Zdaniuk et al., 2011) in DS brains. However, few studies have explored the cause and consequence of T21-dependent changes in astrocytes.

Protocols to differentiate iPSCs to astrocytes using growth factor-mediated approaches begin by differentiating to the NPC lineage, followed by addition of specific growth factors such as bone morphogenic protein 4 (BMP4) and ciliary neurotrophic factor (CNTF) to induce the astrocytic lineage. Early methods were capable of generating iPSC-derived cells that expressed a relatively high level of astrocyte markers [i.e., glial fibrillary acid protein (GFAP) and S100ß] and exhibited functional characteristics of astrocytes such as spontaneous calcium transients, ability to uptake glutamate, and stimulation-dependent cytokine secretion (Krencik et al., 2011; Emdad et al., 2012; Shaltouki et al., 2013). However, these protocols were plagued by extended culture periods (>180 days), scale-up challenges, and/or lack the characteristic star-shaped morphology that astrocytes exhibit in the brain milieu (Voulgaris et al., 2022). Overexpression of the glial lineage transcription factors SOX9, NFIA and/or NFIB in iPSCs was capable of generating induced astrocytes (iAstrocytes) in weeks compared to months (Canals et al., 2018; Li et al., 2018; Tchieu et al., 2019; Neyrinck et al., 2021).

To date, there have only been a handful of studies that have differentiated DS iPSCs to the astroglial lineage, and all studies utilized passive or factor-mediated differentiation techniques. T21 iPSC-derived astrocyte-like cells generated using these methods exhibited a toxic effect on neurons in both euploid and T21 co-culture and media carry-over systems (Chen et al., 2014; Araujo et al., 2018; Mizuno et al., 2018) (Table 1), suggesting that astrocytes may play a role in neuronal dysfunction in DS. Two of the studies focused on S100ß since it is highly expressed in astrocytes and the gene is encoded on chromosome 21 (Chen et al., 2014; Mizuno et al., 2018). Indeed, *S100ß* was found to be upregulated in T21 astrocyte-like cells and modulation of *S100ß* levels was able to restore specific phenotypes including elevated reactive oxygen species and abnormal calcium signaling (Chen et al., 2014). However, a subsequent study demonstrated extensive genome-wide transcriptional alterations due to trisomy 21 in iPSC-derived astrocyte-like cells (Ponroy Bally et al., 2020), indicating that astrocyte phenotypes likely result from dysregulation of a number of genes. Intriguingly, they identified altered expression of numerous cell adhesion and extracellular matrix (ECM) components, which has been observed in a number of DS cell types including iPSC-derived NPCs (Meharena et al., 2022) and neurons (Gonzales et al., 2018; Huo et al., 2018), and found that DS iPSC-derived astrocytes exhibit increased migratory ability (Ponroy Bally et al., 2020) (Table 1). Finally, a recent study used the elegant *XIST* silencing approach pioneered in Jeanne Lawrence’s lab (Jiang et al., 2013) to examine the impact of silencing the third copy of chromosome 21 in trisomic cells on iPSC-derived astrocyte-like cells, which they termed astrocyte precursor cells (APCs) (Kawatani et al., 2021). They observed elevated proliferation of APCs and used subtractive transcriptome analysis to uncover that *DYRK1A* and *PIGP*, two genes on HSA21, are critical regulators of APC proliferation.

While studies have begun to address the underlying cause of elevated astrocyte number in the DS brain, we still lack a detailed understanding of the functional consequences of T21 in astrocytes. Advances in differentiation methods and co-culture systems will inevitably provide insight into the role of astrocytes in DS, as well as the impact of T21 astrocytes on other cell types such as neurons, microglia, and oligodendrocytes.

## iPSC-derived oligodendrocytes

Myelination is performed by oligodendrocytes, whose primary role is to produce the myelin sheath that insulates the axons of nerve cells and forms the white matter of the central nervous system (Simons and Nave, 2015; Dimou and Simons, 2017). Development and maturation of the white matter is critical for proper neuronal circuit function and is correlated with cognitive function and increased motor skills (Nagy et al., 2004). Individuals with DS display reduced white matter content that presents as a delay in the onset of myelination (Wisniewski and Schmidt-Sidor, 1989) and reduced density and disorganization of myelin fibers (Ábrahám et al., 2012; Olmos-Serrano et al., 2016). Despite this clinical phenotype, very few studies have investigated myelination in the context of DS. Therefore, studying DS iPSC-derived oligodendrocyte progenitors and myelinating oligodendrocytes may provide important clues as to the mechanistic basis for white matter anomalies in individuals with DS.

In recent years, stem cell models of oligodendrocyte differentiation and maturation have enabled studies of the mechanisms underlying human oligodendrocyte differentiation, establishment of myelination, and myelin maintenance. Similar to astrocyte differentiation, growth factor-mediated oligodendrocyte differentiation protocols begin with iPSC-derived NPCs that are patterned with SHH and RA, resulting in upregulation of OLIG2 and NKX2.2. Those NPCs are then exposed to factors known to drive oligodendrocyte differentiation such as platelet-derived growth factor (PDGF-AA), neurotrophin 3 (NT3), triiodo-L-thyronine (T3), insulin-like growth factor 1 (IGF-1), and hepatocyte growth factor (HGF) to generate oligodendrocyte progenitor cells (OPCs) (Douvaras and Fossati, 2015). Similar to astrocyte induction protocols, oligodendrocyte differentiation protocols suffer from long culture periods (>2.5 months) and results in a heterogeneous population of cells that requires selection methods to purify OPCs. To enable more rapid generation of oligodendrocytes, some groups have utilized transcription factor-mediated overexpression (i.e., SOX10, NKX6.2, NKX2.2, and OLIG2) approaches to generate myelination-capable oligodendrocytes in approximately 1 month (Ehrlich et al., 2017). Regardless, the process of myelination requires a neuronal substrate, therefore necessitating neuron-oligodendrocyte co-cultures. Alternatively, artificial scaffolds that resemble axons have enabled myelination studies without neurons (Lee et al., 2013; Mei et al., 2014; Espinosa-Hoyos et al., 2018).

A recent study identified that T21 NPCs exhibit dysregulation of SHH signaling that resulted in increased proportions of OLIG2^+^ progenitors and reduced NKX2.2^+^ cells (Klein et al., 2021). As NKX2.2 is a transcription factor that specifies oligodendrocyte lineage cells (Qi et al., 2001), and OLIG2^+^ progenitors can specify either interneurons or oligodendrocytes (Petryniak et al., 2007; Xu et al., 2019), these results could indicate the moleculr basis of impaired oligodendroglial fate at the expense of elevated interneuron specification in DS (Klein et al., 2021). This finding is consistent with the clinical phenotype of reduced myelination (Wisniewski and Schmidt-Sidor, 1989; Ábrahám et al., 2012; Olmos-Serrano et al., 2016) and altered proportions of neuronal subtypes (Chakrabarti et al., 2010; Guidi et al., 2018; Huo et al., 2018; Xu et al., 2019; Stagni et al., 2020) in individuals with DS. However, further studies in utilizing DS iPSCs to examine mechanisms of fate specification in NPCs, OPC differentiation, oligodendrocyte myelination, as well as the contribution of other cell types to this process will help to delineate the impact of T21 on myelination and myelin maintenance.

## iPSC-derived microglia

Microglia are the resident innate immune cells of the brain. They continuously sense and respond to their environment to maintain homeostasis and accomplish highly specialized functions such as phagocytosis, antigen presentation, and cytokine release to regulate several critical aspects of brain development, maturation, and function (Paolicelli et al., 2022). Microglia development occurs in synchrony with the developing brain, however their origin is distinct from other brain cell types in that their progenitors originate from primitive hematopoiesis in the embryonic yolk sac (Ginhoux et al., 2010). Microglia precursors migrate from the yolk sac and infiltrate the developing brain prior to the onset of neurogenesis [∼E8.5 in the mouse (Navascués et al., 2000; Ginhoux et al., 2010); ∼4 gw in human (Menassa et al., 2022)]. During the embryonic period, microglia play active roles in regulating brain development through secretion of cytokines and phagocytosis of excess progenitor cells. In the early postnatal period, microglia regulate synapse development and circuit formation through activity-dependent synaptic pruning, and later, exhibit surveillant functions to maintain homeostasis. In aging and neurodegeneration, microglia shift away from the homeostatic surveillant state and begin to exhibit pathological characteristics such as altered phagocytosis and synaptic engulfment, dysfunctional regulation of myelination, and release of pro-inflammatory cytokines (Chen and Colonna, 2021). This shift has been observed in both mouse models and human postmortem tissue samples, however, the exact genes that define the transcriptional signature of aging- or neurodegeneration-associated microglia are different between the two species (Chen and Colonna, 2021). Moreover, studies point to differences between human and mouse microglia in terms of their transcriptional profile (Galatro et al., 2017; Gosselin et al., 2017; Geirsdottir et al., 2019), highlighting the importance of studying human microglia in the context of human neurological disorders.

Several methods have been developed to differentiate iPSCs into microglia-like cells (iMGLs) (Muffat et al., 2016; Abud et al., 2017; Douvaras et al., 2017; Haenseler et al., 2017). These protocols rely on differentiating iPSCs to mesodermal lineage cells that resemble primitive hematopoietic progenitors using cytokines such as vascular endothelial growth factor (VEGF), stem cell factor (SCF), Fms related receptor tyrosine kinase 3 (Flt3), and/or thrombopoietin (TPO). Once hematopoietic lineage cells (microglia progenitors) are generated (approximately 1.5 weeks), medium supplemented with IL-34 and mCSF induces differentiation to iMGLs that express markers such as CX3CR1, IBA1, and P2RY12 within 1 month. While iMGLs exhibit functional characteristics of microglia such as phagocytosis and synaptic pruning, their transcriptional signature is distinct from that observed in human microglia freshly isolated from the brain (Hasselmann et al., 2019). This *in vitro* transcriptional effect in human microglia had been observed previously, as microglia maintained outside of the brain environment display global transcriptional alterations on the order of hours after transfer to culture conditions (Gosselin et al., 2017). Since these discoveries, several groups have utilized organoid culture or human-mouse chimera systems to enhance microglia differentiation and better recapitulate *in vivo* morphological and transcriptional signatures (Hasselmann et al., 2019; Mancuso et al., 2019; Svoboda et al., 2019; Xu et al., 2020). Injection of microglia progenitors into the brain of neonatal transgenic immunocompromised mice that express a human mCSF gene evades immunogenic rejection of the grafted cells and results in differentiation of xenotransplanted microglia (Abud et al., 2017; Hasselmann et al., 2019; Svoboda et al., 2019; Xu et al., 2020) (xMG; reviewed further in the Human-Mouse Chimera section below). Using this strategy, a recent study demonstrated that DS microglia engage in enhanced synaptic pruning, resulting in impaired neurotransmission in the chimeric DS mice relative to mice engrafted with euploid microglia (Table 1). Exposure of DS microglia to pathological hyperphosphorylated tau resulted in dystrophic phenotypes including process beading and ferritin immunoreactivity, similar to what has been observed in DS-AD (Streit et al., 2009; Xue and Streit, 2011) (Table 1). This study suggests that enhanced synaptic pruning in DS may be, at least in part, due to aberrant phagocytosis of synapses.

## iPSC-derived 3D models

The brain is a complex structure composed of diverse cell types that work together to establish the brain during development and to maintain proper brain function. While relatively homogeneous cultures of specific brain cell types as those described above provide a wealth of information related to the cell autonomous functions (or dysfunctions) that occur in disorders such as DS, they fail to capture a complete picture of the cell-cell interactions that occur in the brain environment throughout the course of neurological disease. To overcome these limitations, more complex models of the brain are in development, including cerebral and brain-region specific organoids as well as human-mouse chimeras.

Cerebral organoids were first described a decade ago that took advantage of the propensity of human pluripotent stem cells grown in suspension aggregates to preferentially differentiate into neuroectoderm in the absence of patterning factors (Kadoshima et al., 2013; Lancaster et al., 2013; Paşca et al., 2015). This method generates a huge variety of brain cell types from different regions in addition to retina, choroid plexus, and mesodermal lineage cells. However, the stochastic nature of the protocol renders it vulnerable to a high degree of variability between organoids, making comparative analysis challenging. Alternative methods to generating organoids utilize patterning factors in a similar manner to 2D differentiation approaches: first, dual SMAD inhibition is typically used to specify iPSCs towards a neural lineage, followed by fine-tuning of region-specific morphogens. Brain development occurs along the dorsoventral and rostrocaudal axes. The neuroectoderm forms the neural tube and develops along the rostrocaudal axis into the prosencephalon (forebrain), mesencephalon (midbrain), rhombencephalon (hindbrain), and spinal cord. RA, WNTs, and fibroblast growth factors (FGFs) cause caudalization, while their inhibition promotes rostral differentiation. The morphogen SHH is critical for ventralization and BMPs and WNTs are necessary for dorsal fate patterning. These same cues are used to generate brain region-specific organoids.

The majority of brain organoid studies utilize methods that mimic the composition of the dorsal forebrain, resulting in ventricular-like neural rosette structures that differentiate into neurons and recapitulate the inside-out migration of the six-layered cerebral cortex. However, as oligodendrocytes and interneurons primarily originate from the ventral forebrain, their numbers are limited in these models. Activation of SHH signaling can induce ventralization of NPCs and organoids (Maroof et al., 2013; Nicholas et al., 2013) and incorporation of growth factors such as PDGF-AA and IGF-1 can help to promote the oligodendroglial lineage (Hubler et al., 2018; Madhavan et al., 2018). However, given that microglia are not of neuroectodermal origin, they must be exogenously added into the patterned systems in order to generate organoids with all major brain cell types. Microglia have been reported in unguided organoids (Ormel et al., 2018), likely arising from mesodermal progenitors that are found in variable number from batch to batch (Quadrato et al., 2017). However, the inconsistency in mesodermal progenitor frequency and thus microglia frequency across individual organoids remains an issue.

In the context of DS, several brain region-specific organoids are of interest to gain insights into the regional vulnerability of the disorder including dorsal and ventral forebrain, hippocampal, retinal, and cerebellar organoids. Cerebral organoids generated from DS iPSCs display pathological AD hallmarks including elevated amyloid-ß and hyperphosphorylated tau (Gonzalez et al., 2018; Zhao and Haddad, 2022; Campbell et al., 2023; Czerminski et al., 2023) (Table 1). Beyond this phenotype, DS studies have also been susceptible to organoid variability, making the identification of phenotypic differences between DS and euploid organoids challenging (Li et al., 2022; Czerminski et al., 2023). Despite this, DS organoids tend to be smaller than those generated from neurotypical iPSCs (Tang et al., 2021; Li et al., 2022; Campbell et al., 2023) and single-cell RNA-seq suggested altered proliferation and delayed neurogenesis (Tang et al., 2021) as well as altered excitatory neuron production, particularly of layer IV neurons (Tang et al., 2021; Li et al., 2022) (Table 1). Further, electrophysiological characterization of organoids generated from T21 iPSCs indicated that while DS organoids were capable of generating signatures of mature neuronal activity by 6–10 months in culture, they displayed reduced neuronal network communication, altered neuronal excitatory-to-inhibitory balance, and dysfunctional GABAergic neuronal activity (Foliaki et al., 2021) (Table 1).

By generating ventralized organoids, Xu et al. (2019) presented evidence that OLIG2^+^ progenitor cells, which specify GABAergic interneuron fate, are elevated in frequency in T21 organoids. This increase in fate-restricted progenitors was correlated with an increased frequency of specific interneuron subtypes including calretinin- (CR), somatostatin- (SST), and GAD65/67-expressing cells in both ventralized organoids and postmortem DS brain tissue (Xu et al., 2019). Normalization of OLIG2 levels in T21 organoids restored GABAergic neuron production to control levels (Xu et al., 2019), suggesting that OLIG2 may be a key factor in driving elevated interneuron production in DS. However, in depth exploration of the molecular mechanisms governing this phenomenon remain unexplored.

Although brain organoid technology has made incredible advances in the past decade, these models still lack some key components. In particular, the complex cerebrovascular network that contributes to formation of the blood-brain barrier (BBB) has proven challenging to recapitulate in 3D cultures, but this type of system could provide significant insights into the cerebrovasculature changes observed in DS (Head et al., 2017). The BBB is composed of brain endothelial cells, pericytes, and astrocytes (Abbott et al., 2006; Daneman and Prat, 2015). Current techniques are capable of differentiating the individual BBB cell types *in vitro* from iPSCs (Tcw et al., 2017; Neal et al., 2019; Aisenbrey et al., 2021) and protocols to generate blood vessel organoids have been described (Werschler and Penninger, 2023). Advanced bioengineering approaches such as microfluidic organ-on-a-chip (Vatine et al., 2019) and synthetic scaffolding (Robert et al., 2017) have successfully built perfusable BBB models, yet the diameter of the vessels are orders of magnitude larger than what is observed *in vivo*, calling into question how well they truly model the BBB. Some groups have also used an assembloid-like strategy to combine brain organoids with blood vessel organoids to accomplish brain organoid vascularization complete with microglia integration from the mesodermal patterning of blood vessel organoids (Sun et al., 2022). While this strategy was successful in generating a complex 3D model of the brain, it still lacks the tube-like structures of blood vessels and the ability to model active blood flow.

## Human-mouse chimeras

Despite the promise of organoid technology to provide improved model systems to study human brain development and disease, a major shortcoming is the lack of a behavioral read-out. To circumvent this limitation, groups have turned to human-mouse chimera systems that enable engraftment of human stem cell-derived cell types into the mouse brain. Immunodeficient SCID mice or mice lacking recombination activating gene 1 or 2 (Rag1^−/−^ or Rag2^−/−^) are used to prevent host rejection of the graft, and in some cases, such as for microglia differentiation, additional human transgenes are necessary to support survival and differentiation of the human cells (Abud et al., 2017; Hasselmann et al., 2019).

Studies utilizing xenotransplantation of DS iPSC-derived brain cells into immunodeficient mice (Rag1^−/−^, Rag2^−/−^, or SCID) have successfully generated human-mouse chimeras engrafted with cortical excitatory neurons (Real et al., 2018), GABAergic interneuron progenitors (Huo et al., 2018), microglia precursors (Jin et al., 2022) (reviewed in the Microglia section), and ventralized NPCs (Xu et al., 2019) (Table 1). Real et al. (2108) grafted a mixed population of fluorescently-labeled iPSC-derived cortical neuronal progenitors and neurons into the somatosensory cortex of adult mice and used two-photon imaging to monitor the cells over time (Aisenbrey et al., 2021). Transplanted DS neuronal cells generated progenitors, neurons, and proliferating cells to a similar extent as controls, however, DS progenitors preferentially differentiated into astroglia (Aisenbrey et al., 2021), consistent with human observations (Guidi et al., 2008). Neuronal synaptic development was similar, yet longitudinal *in vivo* imaging showed increased synaptic stability and altered neural population activity as measured by calcium imaging in DS cortical neurons (Aisenbrey et al., 2021). Huo et al. (2018) transplanted GABAergic precursors into the medial septum of 8 to 10-week old mice and found defects in interneuron differentiation, migration, and ability to project axons. A subsequent study engrafted 5-week-old ventralized organoid cells, which primarily consist of FOXG1^+^NKX2.1^+^ NPCs, into the cortex of neonatal pups and recapitulated *in vitro* findings of increased production of GABAergic neurons (Xu et al., 2019). Mice engrafted with DS interneuron progenitors displayed impaired recognition memory relative to non-engrafted mice (Xu et al., 2019). It remains unclear whether this specific behavior is related to T21-induced alterations in interneurons that integrated into the mouse brain circuitry, or if interneuron xenotransplants induce this behavioral deficit regardless of genotype.

Collectively, the chimeric system enables detailed study of the cell autonomous consequences of T21 on human brain cell types in a living organism as well as behavioral read-outs of cell type-specific consequences of T21. To properly interpret rodent behavioral assays, it is critical to first assess the overall health of the animal. Compromised vision has been reported in the Rag2^−/−^ immunocompromised mouse strain (Han et al., 2013), suggesting that this system may not be appropriate for behavioral tests requiring a visual component. Some additional caveats of human-mouse chimeras include residual host cell types and lack of a peripheral adaptive immune system in immunocompromised mice. The former issue is challenging to overcome, yet for cell types such as microglia that can be easily depleted using pharmacological (Elmore et al., 2014) or genetic (Rojo et al., 2019) means, it is possible to completely deplete host cells while simultaneously engrafting the brains with human microglia precursors (Chadarevian et al., 2022). It is also reasonable to engineer an adaptive immune system from the same human donor in the immunodeficient mice, providing a system to study the relationship between the peripheral immune system and the brain in development and disease (Li et al., 2017; Jiang et al., 2020).

More recently, groups have transplanted whole organoids into the early postnatal rodent brain and observed functional integration into relevant neural circuits (Revah et al., 2022; Jgamadze et al., 2023). Transplanted organoids showed evidence of vascularization with rat endothelial cells and exhibited more mature properties than *in vitro* organoids such as increased growth, higher synaptic density, elevated neuronal activity, and a slightly more advanced transcriptional signature (Revah et al., 2022). Importantly, activation of these human neurons through optogenetic or visual stimulation can drive specific behaviors in the chimeric animal, paving the way for disease-relevant studies to detect circuit-level phenotypes using patient-derived organoids.

## Conclusion and future directions

The brain is one of the most impacted organs in response to trisomy 21 and neurological dysfunction is a primary contributing factor to reduced quality of life in individuals with DS. Human postmortem studies have indicated some consistent clinical features of the DS brain, including reduced volume, dysfunctions in NPC proliferation and differentiation, altered neuronal connectivity and communication, and neuroinflammation. Some of these features have been successfully modeled using rodents, however, species-specific differences in brain development and maturation preclude our ability to fully model neurological disorders like DS and hinder the development of effective therapeutics.

It has been 15 years since the generation of iPSCs from individuals with DS, which were among the first disease-associated iPSC lines to be reported (Park et al., 2008b). Since then, advances in neurological disease modeling with human stem cells have enabled mechanistic studies of the pathophysiology of neurological diseases. We are now capable of differentiating iPSCs into all of the primary brain cell types, and co-culture and 3D organoid systems are emerging that permit study of the complex interactions between different brain cells and regions. As these methods continue to evolve, our ability to mimic *in vivo* conditions and model neurological diseases such as DS and DS-AD will certainly improve.

To date, DS iPSC-derived brain cell types have recapitulated NPC dysfunctions including altered proliferation and differentiation, implicating key signaling pathways such as WNT (Giffin-Rao et al., 2022) and SHH (Klein et al., 2021), specific genes encoded on chromosome 21, and disorganization of higher-order chromatin architecture (Meharena et al., 2022) as mechanisms underlying this phenotype. Several questions remain, including the exact cause and consequence of altered NPC proliferation, skewed differentiation trajectory of T21 NPCs, and T21-induced NPC senescence. Groups have begun to explore the use of cerebral organoids and other 3D culture systems in DS, which will likely provide a good platform to address these and other questions, such as the impact of T21 on astrocytes, oligodendrocytes, and microglia as well as the interaction between these cell types and NPCs/neurons in the context of DS. In the future, brain region-specific organoids, assembloids, and brain-on-a-chip technologies can be used to investigate regional vulnerability in DS by focusing on the structures (i.e., hippocampus and cerebellum, in addition to the cerebral cortex) and circuits particularly impacted by T21.

While significant advances have been made in 3D cell culture techniques, these systems are still unable to fully recapitulate the cellular diversity, developmental niches, and regional structures that exist in the brain. For instance, while protocols exist to generate hippocampal organoids, they lack formation of the neurogenic niche thus hindering adult neurogenesis studies in this system. Further, there is a growing appreciation for the enormous cellular diversity that exists in the brain, first described in Ramon y Cajal’s seminal work (Cajal et al., 1995). Single-cell approaches are uncovering a wide range of distinct cell type-specific molecular states *in vivo* over the lifespan and in disease that will undoubtedly serve as a reference to facilitate generation of these cell states *in vitro* and refinement of culture systems to study the cellular and molecular mechanisms underlying neurological disorders such as DS.
